# Dual tracer navigation for lymph node dissection in laparoscopic radical gastrectomy (DANCE trial): a protocol for a prospective, randomized clinical trial

**DOI:** 10.1186/s13063-023-07676-4

**Published:** 2023-10-02

**Authors:** Yanjun Lu, Shichao Ai, Peng Song, Yan Sun, Xiaofei Shen, Feng Sun, Qiongyuan Hu, Zhiyan Li, Meng Wang, Xiaofeng Lu, Wenxian Guan, Song Liu

**Affiliations:** 1 Division of Gastric Surgery, Department of General Surgery, Nanjing Drum Tower Hospital, the Affiliated Hospital of Nanjing University Medical School, Nanjing, China; 2 Department of Anesthesiology, Nanjing Drum Tower Hospital, the Affiliated Hospital of Nanjing University Medical School, Nanjing, China

**Keywords:** Lymph node, Indocyanine green, Carbon nanoparticles, Gastric cancer

## Abstract

**Background:**

Lymph node (LN) metastasis is the most common metastasis route in gastric cancer. Extensive dissection of LNs can significantly improve the prognosis of patients with gastric cancer. Recently, multiple clinical studies have demonstrated that either indocyanine green (ICG) or carbon nanoparticles (CNs) can assist to promote the dissection of LNs during laparoscopic radical gastrectomy. Considering the pros and cons of the two tracers, this study proposed a novel method of dual tracer (ICG combined with CNs) for lymphatic tracing in laparoscopic gastric cancer surgery.

**Methods:**

This trial is a prospective, randomized controlled trial (RCT) with an estimation of 516 participants that randomize into 4 groups (1:1:1:1), namely control group, ICG group, CNs group, and dual tracer group. The primary outcome is the number of dissected LNs. The secondary outcomes include positive rate, false positive rate, negative rate, false negative rate, number of metastatic LNs, relationship between LN metastasis and tracer stained, operation duration, blood loss, incision length, morbidity and mortality rate, 3-year DFS (disease free survival), PFS (progression-free survival), and OS (overall survival).

**Discussion:**

This study will investigate the efficacy and safety of a novel strategy using dual tracers for laparoscopic gastrectomy. The protocol has been approved by the Ethics Committee of Nanjing Drum Tower Hospital (2021-361-02). The trial findings will be published in peer-reviewed journals.

**Trial registration:**

Chinese Clinical Trial Registry (ChiCTR2100051309). Registered 18 September 2021, https://www.chictr.org.cn/showproj.html?proj=133764.

**Supplementary Information:**

The online version contains supplementary material available at 10.1186/s13063-023-07676-4.

## Introduction

Lymph node (LN) metastasis is the most common mode of metastasis in gastric cancer. Even in early gastric cancer, the rate of LN metastasis is about 2–20% [1]. Numerous studies have shown a close correlation between number of dissected LNs and accuracy of postoperative pathological staging and long-term prognosis [2, 3]. However, it is a great challenge for surgeons to identify LNs from hypertrophic adipose tissue or complex lymphoid tissue without increasing the risk of surgery and postoperative complications. Therefore, improving intraoperative visualization of LNs becomes an urgent clinical issue [4].

In recent years, navigation technique using tracers such as indocyanine green (ICG) and carbon nanoparticles (CNs) to visualize LNs has been proved to be effective for sentinel LN tracing and dissection in breast cancer, lung cancer, and other tumors [5–10]. Also, it has been confirmed that either ICG or CNs can assist surgeons to dissect LNs in laparoscopic gastrectomy. Nevertheless, there are still limitations in the application of two tracers.

ICG administration includes multi-point subserosal injection and peritumor submucosal injection [9, 11–13]. Subserosal injection of ICG can help to obtain better LN navigation, but it also usually leads to the leakage of fluorescence and spilled the surgical field [13]. Submucosal administration of ICG helps tumor localization but restrains LN visualization compared to subserosal administration.

CNs is mainly administrated by peritumoral submucosal injection under preoperative gastroscopy. CNs can induce satisfactory tumor localization due to its intuitive black staining effect and rarely brings the contamination of surgical fields. However, submucosal injection of CNs is inferior in lymphangiography because of its limited retention capacity in lymphatic vessels. In addition, the accumulation of CNs in distant LNs is much less than that in peritumoral LNs [14, 15]. Collectively, single tracer (either ICG or CNs) strategy demonstrates unfavorable technical bottlenecks. We notice that the disadvantages of ICG tracer technology can be complemented by the advantages of CNs tracer technology, and vice versa. Consequently, it is promising to implement dual tracers into laparoscopic gastrectomy for a synergistic effect in LN visualization.

## Methods

### Study design

**Fig. 1:**
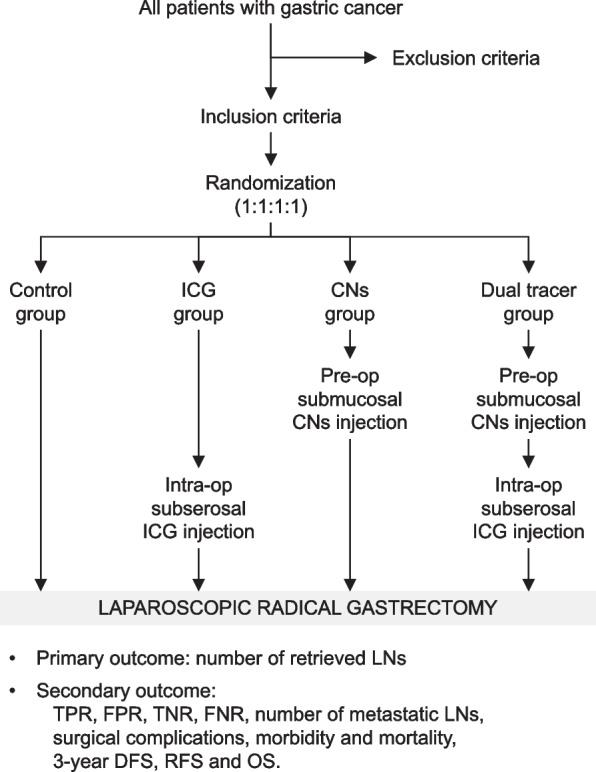
Flow chart of this study

**Fig. 2:**
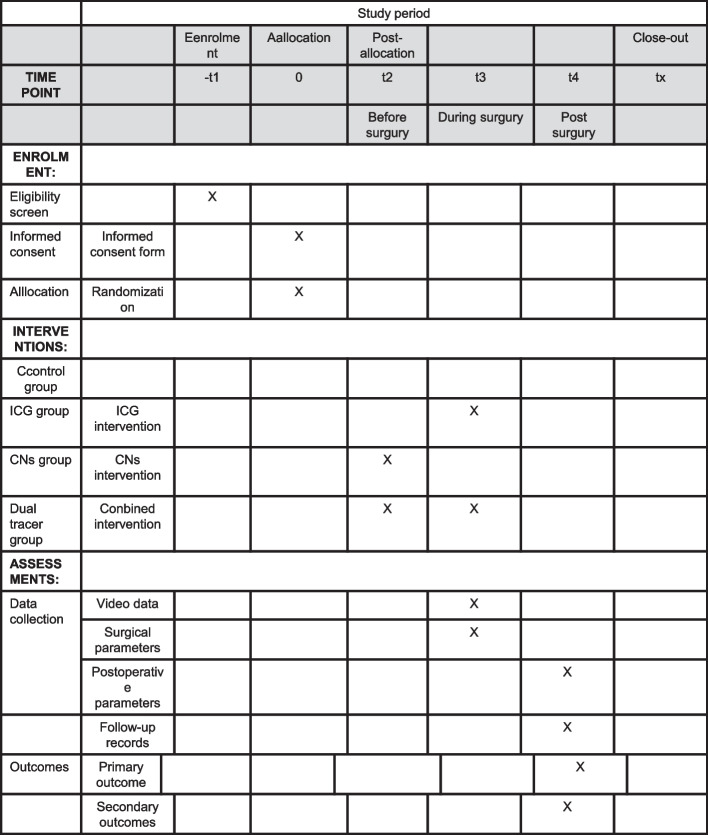
Content for the schedule of enrolment, interventions, and assessments

This is a prospective, unblinded, superiority testing, randomized controlled trial. A parallel group cluster randomized controlled trial with four arms will be conducted. Figure 1 demonstrates the flow chart of current study. Figure 2 contents the schedule of enrolment, interventions, and assessments. Participants were randomized into 4 groups at a 1:1:1:1 ratio: (1) control group: participants will undergo conventional laparoscopic radical gastrectomy without any tracer; (2) ICG group: participants will undergo laparoscopic radical gastrectomy with subserosal ICG navigation; (3) CNs group: participants will undergo laparoscopic radical gastrectomy with submucosal CNs navigation; (4) dual tracer group: participants will undergo laparoscopic radical gastrectomy with both tracers, i.e., subserosal ICG and submucosal CNs. Primary and secondary outcomes will be assessed after the gastrectomy. The study protocol is reported in accordance with the guidelines outlined in the SPIRIT Checklist for Trials (Additional file 1) [16].

### Study setting

This study is a single-center trial that will be conducted in the Division of Gastric Surgery, Department of General Surgery, Nanjing Drum Tower Hospital, the Affiliated Hospital of Nanjing University Medical School. This hospital is a large comprehensive tertiary healthcare institute that attracts patients from Jiangsu, Anhui, and other provinces in China.

### Sample size

This study is a superiority testing trial (one-tailed trial) of which the primary outcome is the number of retrieved LNs and study subjects will be divided into 4 groups. Combined with our previous findings and literature reports, the standard deviation σ of the total number of LNs in control group was 8.6 [9, 13, 17]. The *α* (test level) is set as 0.05 and power (1-β) is 0.8. A difference of more than 3 in the total number of detected LNs will be considered as significant, i.e., the effect value *d* = 3/*σ* = 0.35. Thus, at least 103 (102.3) participants are required in each group. Considering an expected dropout rate of 20%, 129 (128.75) participants are finally required in each group, leading to a total of 516 participants.

### Participants

#### Inclusion criteria

The following are the inclusion criteria: (1) 18–75 years old; (2) primary gastric adenocarcinoma (papillary, tubular, mucinous, signet ring, undifferentiated adenocarcinoma) confirmed pathologically by endoscopic biopsy before surgery; (3) cT1-4a, cNany, cM0 according to the AJCC classification (version 7.0) before surgery; (4) absence of adjacent organ invasion and distant metastasis; (5) ECGO score 0 or 1 and ASA grades I–III; (6) signed informed consent.

#### Exclusion criteria

The following are the exclusion criteria:(1) pregnant or lactating women. (2) serious mental disease; (3) previous upper abdominal surgery (except laparoscopic cholecystectomy); (4) previous gastric surgery (including ESD and EMR); (5) refusal to laparoscopic surgery; (6) allergy to iodinated contrast agents; (7) peri-gastric LN larger than 3 cm in preoperative imaging; (8) history of other malignancies within recent 5 years; (9) preoperative neoadjuvant chemoradiotherapy; (10) unstable angina pectoris or myocardial infarction within recent 6 months; (11) continuous systemic use of hormones within recent 1 month; (12) combined with other surgeries (except appendectomy or cholecystectomy); (13) emergent surgery due to complications caused by gastric cancer (bleeding, perforation or obstruction); (14) FEV1 less than 50% of the predicted value; (15) conversion to laparotomy.

#### Withdrawal criteria

The following are the withdrawal criteria: (1) diagnosis of M1 during or after surgery, i.e., distant metastasis upon intraoperative exploration or postoperative pathology, or positive exfoliated cells in the abdominal cavity; (2) duodenum invasion of diagnosis of T4b during or after surgery; (3) incomplete D2 dissection or R0 resection owing to masses formed by regional LNs coalescing or vital vessel surrounding; (4) necessary additional surgery during operation; (5) serious perioperative complications (unable to tolerate surgery or anesthesia); (6) referring to emergent surgery due to condition changes judged by attending physicians; (7) voluntary withdrawal or discontinued treatment due to personal reasons at any stage; (8) treatment violates study protocol.

### Recruitment

The clinical investigators will be trained on comprehensive assessment of inclusion, exclusion and withdrawal criteria. Every patient diagnosed with gastric cancer in the hospital will be screened for qualification. The investigators will communicate with potential participants and their relatives. In this way, potential participants will be recruited professionally. Recruitment progress will be monthly summarized and reported to the trial director to ensure sufficient recruitment of participants. The expected recruitment rate is 20–22 participants per month, and the recruitment progress is expected to last for 2 years.

### Randomization

Participants will be randomized into dual tracer, ICG, CNs, or control (non-tracing) group. A random digit table is used for randomization as follows: (1) according to the order of inclusion, each participant will be allocated a random number, and (2) divide the number by 4. If the remainder is 0, the participant will be enrolled into dual tracing group. If the remainder is 1, the participant will be enrolled into non-tracing group. If the remainder is 2, the participant will be enrolled into CNs group. If the remainder is 3, the participant will be enrolled into ICG group. The allocation is not blinded to surgeons or participants before performing procedure.

### Outcome

The primary outcome is the total number of retrieved LNs. The secondary outcomes include positive rate, false positive rate, negative rate, false negative rate, number of metastatic LNs, rate of LN metastasis, relationship between LN metastasis and dual staining, morbidity and mortality, 3-year disease-free survival rate, 3-year overall survival rate, 3-year recurrence rate, postoperative recovery, duration of surgery, changes in body weight, intraoperative blood loss, intraoperative mortality, incision length, body temperature, and changes in lab parameters (albumin, prealbumin, hemoglobin, white blood cells, C-reactive protein, etc.).

### Eligibility of surgeons

An experience of at least 100 laparoscopic radical gastrectomy is required for surgeons involved in this study.

### Intervention

#### Preoperative administration of CNs

Following enrollment, surgery should be performed within 2 weeks (including day 14). In addition to routine preoperative management, peritumoral submucosal CNs injection is administered 1 day before surgery in CNs group and dual tracer group. Carbon nanoparticles suspension injection (Chongqing Lummy Pharmaceutical Co., Ltd.) is diluted into 25 mg/ml with saline and then injected using “sandwich method.” Briefly, approximately 0.5 ml saline is firstly injected into the submucosal layer for mucosal elevation. Then, 0.2 ml CNs is injected. Finally, 0.5 ml saline is injected again to prevent leakage of CNs. This injection is performed at both proximal and distal sides of tumor under gastroscopy.

#### Intraoperative lymph node localization and dissection

After anesthesia, ICG (Dandong Yichuang Pharmaceutical Co., Ltd.) powder is diluted into 2.5 mg/ml with saline and then injected into subserosal layer using “hexa-site” method in the ICG group and dual tracer group [18]. Briefly, the injection is performed at 3 sites along the lesser curvature (first branch of left gastric artery, angular and first branch of right gastric artery). Similar injection was repeated along the greater curvature.

A near-infrared (NIR) fluorescence imaging system (PINPOINT, NOVADAQ, Mississauga, ON, Canada) is used to obtain near-infrared fluorescence imaging during surgery. It provides high-definition white light visual field, near-infrared fluorescence visual field, and ICG-specific SPY visual field as well as merged visual field. LN dissection in the ICG group and dual tracer group is performed under the fluorescent laparoscope.

The standard sequence of LN dissection is as follows: (1) total gastrectomy: No. 6 → No. 7, 9, 11p → No. 8a, 12a, 5 → No.1 → No. 4sb → No. 4sa, 11d → No.2; (2) distal subtotal gastrectomy: No. 6 → No. 7, 9, 11p → No. 3, 1 → No. 8a, 12a, 5 → No. 4sb. Selective dissection of No.10 LNs is performed when (1) the primary tumor is located in the middle/upper stomach and invaded a large extent, (2) preoperative radiology suggests splenic lymphadenopathy, (3) significant fluorescent signal of No. 10 LNs, and (4) black stained No. 10 LNs by CNs. Surgical area is routinely inspected for possible residual fluorescent LNs or black-stained LNs, and additional dissection will be performed if necessary. D2 + LN dissection will be performed if fluorescent or black-stained LNs (such as No. 10 or No. 14v) are observed outside D2 region.

### Data collection

Data will be collected and entered into the database by clinical investigators and supporting trial personnel on both electronic and paper-based case report forms (CRFs). Specific contents are as follows:

#### Intraoperative data collection

##### Video data

Intraoperative LN dissection and postoperative specimen profile will be photographed and archived for each participant. Furthermore, the entire laparoscopic procedure will be videotaped, and the unclipped files will be saved for evaluation. All photos and videos will be stored in hard drivers for a minimum of 3 years. The procedure will be judged as disqualified if photographs or videos are incomplete.

##### Surgical parameters

The following information will be recorded: (1) name of leading surgeon; (2) procedure duration; (3) procedure type especially digestive tract reconstruction method; (4) incision length; (5) whether converted to open surgery and reason; (6) intraoperative blood loss; (7) blood transfusion volume; (8) tumor location; (9) maximum diameter of tumor size; (10) whether distant metastasis is observed and, if any, the location of metastasis; (11) proximal and distal resection margin, radical cure (R0/R1/R2); (12) intraoperative complications including vascular injury, organ injury, tumor rupture, hypercapnia, emphysema, subcutaneous emphysema, air embolism, allergic reactions, etc.; (13) intraoperative death if occurs.

#### Postoperative parameters

The following information will be recorded: (1) pathological findings including histological type, distant metastasis and location, NIH risk grade, and degree of radical surgery (R0/R1/R2); (2) postoperative complications of which the severity is graded according to the Clavien-Dindo system and serious complication is defined as grade III or above; (3) lab parameters including blood routine test (hemoglobin, red blood cells count, white blood cells count, lymphocyte count, neutrophil count and percentage, platelets count, monocyte count, etc.) and blood biochemistry (albumin, prealbumin, total bilirubin, indirect bilirubin, direct bilirubin, AST, ALT, creatinine, urea nitrogen, total cholesterol, triglyceride, fasting blood glucose, potassium, sodium, chloride and calcium, etc.); (4) postoperative recovery including time to first ambulation, time to first ventilation, time to liquid diet, postoperative highest body temperature, time to gastric tube withdrawal, daily gastric juice drainage volume, time to abdominal drainage tube withdrawal, daily abdominal drainage tube drainage volume, blood transfusion volume from the surgery to discharge, and postoperative hospital stay.

#### Follow-up records

Before discharge, various forms of health education such as dietary brochure and popular science lecture are provided to each participant to clarify the importance of regular follow-up visits. For patient who fails to visit on time for reexamination, telephone contact will be carried out to advise prompt reexamination. All participants will be followed-up every 3 months for the first 2 years and every 6 months since the third year (i.e., 1, 3, 6, 9, 12, 15, 18, 21, 24, 30, and 36 months) after discharge. Table 1 will be filled out at each visit. Tumor recurrence or metastasis and survival status of all participants will be assessed accordingly.

**Table 1:** Follow-up sheet for each participant

Time point since surgery (month)	1	3	6	9	12	15	18	21	24	30	36
Actual date	√	√	√	√	√	√	√	√	√	√	√
Physical examination	√	√	√	√	√	√	√	√	√	√	√
Blood routine	√	√	√	√	√	√	√	√	√	√	√
Blood biochemistry	√	√	√	√	√	√	√	√	√	√	√
Tumor related indicators	√	√	√	√	√	√	√	√	√	√	√
Chest plain scan	√	√	√	√	√	√	√	√	√	√	√
Upper gastrointestinal endoscopy					√				√		√
Abdominal CT			√		√		√		√	√	√
Abdominal ultrasound	√	√				√		√			

### Date management

Electronic CRFs will be stored in a standardized database with periodical backups, and paper CRFs will be submitted to the trial office. Prior to enrollment, clinical investigators and statistical analysts will receive uniform training in data entry, date extraction, and data analysis. The study database will be exposed only to trial staffs who have been trained and authorized. Data analysts will extract data from CRFs and review data for accuracy and completeness. For participants with missing data, missing values will not be supplemented, and the participants will be withdrawn from the study.

Identity and privacy of the participants will be strictly protected. Each enrolled participant will be assigned a subject ID number after signing the informed consent form. This unique ID number will represent the subject’s identity and be entered into the database. Data collected from subject will be stored under the ID number. Several measures will be taken to minimize the disclosure of subjects’ personal information, including (1) only authorized investigators can link the trial data chain of subjects to the subjects themselves through the identification form maintained by the study site, and (2) regulators and supervision departments of this study will conduct on-site review on the original data and strictly keep confidential. Data collection, transfer, processing, and storage will comply with data protection and privacy regulations throughout the study.

### Data analysis

Database will be established using Epidata 3.0, and statistical analysis will be performed using SPSS 18.0. This trial uses differential testing; a *p*-value less than 0.05 will be considered statistically significant, and the confidence intervals for the parameters will be estimated as 95%.

In this trial, validity analysis based on MITT (modified intention to treat) analysis will be conducted. The primary and secondary outcomes will be analyzed with both MITT and PP (per protocol) analysis. Conclusion of MITT analysis will be the primary analytical method, and if the conclusions of PP analysis and MITT analysis are consistent, the credibility of the conclusions can be improved. SP (safety population) analysis will be used to analyze laboratory evaluations, adverse events, and adverse reaction data. SP will be defined as the denominator for the incidence of adverse reactions.

Descriptive statistics will be used to systematically describe primary and secondary outcomes. Means, standard deviations, and confidence intervals will be presented based on data from the control group and intervention groups. Frequency distributions, percentages, medians, and mean rank order will be presented based on quantitative data and rank data. Positive rates, positive number, and total number of retrieved LNs will be presented based on qualitative data. The number of events, number of missingness, median survival time, and survival rate will be presented based on survival data. Efficacy analysis will be performed on data following descriptive statistics. This study utilizes Pearson chi-square test for qualitative data, *t* test for quantitative data, Wilcoxon rank sum test for ranked data, Log-rank test for univariate analysis and Cox regression model for multivariate analysis of survival data, and two-tailed test for safety indicators and incidence of adverse reactions.

Abnormal values will be judged when the observed value is greater than P75 or less than P25, and 3 times more than the interquartile range defined as P75 subtracts P25. Sensitivity analysis will be performed on the abnormal data, and the data will be retained if results are not contradictory. If there is a contradiction, specific analysis will be conducted.

Subgroup analysis will be performed to identify risk factors for the prognosis according to the specific circumstances of the data.

### Oversight and monitoring

#### Date monitoring

The members of the DMC (data monitoring committee) will be independent from the trial sponsor and trial personnel. A senior data statistician will serve as the chairman of the committee, and the rest will be composed of independent data statisticians and clinical scholars. The DMC will hold a Data Monitoring Reporting Meeting in February of each year during the conduct of the trial. In addition to members of DMC, clinical investigators, statisticians, and supporting assistants of the trial will participate in the meeting and provide reports. Data monitoring will be completed by comparing the original data to confirm the completion status of data collection.

#### Adverse event reporting

The evaluation of adverse events and serious adverse events is based on Common Terminology Criteria for Adverse Events v3.0. The study director must report adverse events or serious adverse events to the study committee and the healthcare department of the province (city) according to relevant laws and regulations. Serious adverse events should also be reported to the head of the medical institutions and corresponding reporting procedures shall be completed in accordance with relevant provisions. Adverse events with emergency reporting obligation are as follows: (1) all patients who died during treatment or within 30 days from the last treatment day (regardless of causal relationship with the study protocol treatment) and (2) patients with unexpected grade 4 non-hematological toxicities. Adverse events with regular reporting obligations are as follows: (1) death causally related to treatment 31 days after the last treatment day, including death suspected to be related to treatment (including death caused by obvious primary disease); (2) expected grade 4 non-hematological toxicities; (3) unexpected grade 3 adverse events; (4) other major medical events such as adverse events considered by the trial team to have an potentially, permanent, and significant impact on patients’ offspring. Adverse events in items (2) to (4) above will be reported periodically if any definite or possible causal relationship to the study is identified.

#### Interim analysis

Safety interim analysis and futility interim analysis will be conducted every 8 months, and analysis report will be submitted to the Efficacy and Safety Evaluation Committee. The specific contents of the report include the following: ineligible patients/potentially ineligible patients, reasons for end of treatment/discontinuation/study protocol, adverse events and serious adverse events, completion rate of laparoscopic surgery, proportion of conversion to laparotomy, enrolled protocol deviations, disease-free rate/disease-overall survival rate of all participants, etc. The Efficacy and Safety Evaluation Committee will review and discuss the report in accordance with the procedures documented in the Clinical Safety Information Management Guidelines and make written recommendations to the study director including whether to continue the enrollment of the study.

#### Protocol amendments

The protocol can be amended after discussion and adoption by the study committee meeting, provided that the amendment of the protocol will not cause medical or financial burden or additional risk to enrolled patients. Any changes to the protocol must be identified in writing and signed by the trial director. Significant changes require approval of Institutional Review Board. The updated protocol will be uploaded to the clinical trial registry and copied to clinical investigators.

### Trial status

This is the third version of the protocol. This first version was dated 18 September 2021, and the second version was dated 08 June 2022. Approximate period for recruitment is from 01 August 2023 to 30 July 2025.

## Discussion

Gastric cancer is one of the most common malignancies worldwide, especially in Asia. It has been widely accepted that extensive lymphadenectomy is associated with improve prognosis of patients with gastric cancer. In recent years, navigation surgery using ICG or CNs has been increasingly applied in laparoscopic gastrectomy. Nonetheless, single tracer technique demonstrates certain drawbacks in clinical practice. This study proposes a new strategy using dual tracers (ICG with CNs) for LN visualization in laparoscopic gastrectomy, which is expected to raise the number of retrieved LNs and eventually improve the prognosis of patients.

Dual tracer navigation for lymph node dissection in laparoscopic radical gastrectomy can potentially bring several benefits. Compared with ICG alone, (1) it may reduce false negative rate, which is the most significant limitation of current ICG application; (2) it can optimize the tumor localization; and (3) it can decrease the possibility of surgical field contamination during the leakage of ICG. Compared with CNs alone, (1) lymphatic blockage by tumor cells can affect the distribution of CNs in metastatic LNs. At this point, ICG with smaller molecular weight is able to show lymphatic networks that hardly be reached by CNs, and (2) the combination of the two tracers is beneficial to detect lymph nodes in abnormal anatomical locations and improve lymph node detection rates, leading to accurate pathological staging and treatment plan. All above advantages eventually result in better prognosis of patients with gastric cancer.

### Supplementary Information

**Additional file 1.**

## Data Availability

Data will be made available on reasonable request to the corresponding author.

## Abbreviations

AJCC: American Joint Committee on Cancer staging
ALT: Alanine aminotransferase
ASA: Adenosine sulfate aminotransferase
AST: Aspartate aminotransferase
CNs: Carbon nanoparticles
CRF: Case report form
DFS: Disease free survival
DMC: Data monitoring committee
ECGO: Eastern Cooperative Oncology Group
EMR: Endoscopic mucosal resection
ESD: Endoscopic submucosal dissection
FEV1: Forced expiratory volume in one second
ICG: Indocyanine green
LN: Lymph node
MITT: Modified intention to treat
NIH: National Institutes of Health
OS: Overall survival
PFS: Progression-free survival
PP: Per protocol
RCT: Randomized controlled trial
SP: Safety population
